# Hæmaturia in Quinine

**Published:** 1876-12

**Authors:** G. D. Hodge

**Affiliations:** Prescott, Arkansas


### HÆMATURIA AND QUININE.

By G. D. HODGE, M.D., OF ARKANSAS.

The idea of quinine in abusive doses being the cause, or *one* of the causes, of malarial hæmaturia, first met our observation on perusing an article published in the October number of The Southern Medical Record, of 1874. Subsequently the same position was taken and strongly corroborated with the report of cases by Dr. A. B. Jenkins, of Texas.

It is our lot to practice in a highly malarial district, and ample opportunities have been afforded us of testing the point at issue, and we must say, we have long since been convinced of the truth of the proposition. For ourself, we have yet to see the first case of hemorrhagic fever that did not ensue immediately on a cinchonized condition of the system. Well, admitting that this pernicious form of fever and quinine *do* sustain towards each other the relation of cause and effect, *shall we abandon the use of so potent a remedy* in our treatment? We unhesitatingly answer in the negative. In our field, this fever is always more or less of the congestive or paroxysmal character, and whilst hæmaturia frequently follows the administration of quinine, such is not necessarily the case, but rather *exceptionally* so. Believing, as we do, that an intense malarial poisoning is an essential link in the chain of causes, we never hesitate a moment, but give the quinine; and that, too, in commanding doses, and if hæmaturia supervene, and there still exist a necessity for an antiperiodic and malarial antidote, we immediately suspend this medicine, and substitute in lieu thereof salicine. The latter, we regard as second only to quinine as an antiperiodic tonic and malarial antidote, and on account of its well known alterative effect on mucous surfaces, we regard it as particularly applicable in the management of this disease.

The tinct. gulsemimun, also, we find a valuable auxilliary, conjoined with some suitable diaphoretic, in the febrile stage, and with the quinine or salicine during the remission.

We will only add, since adopting the conclusion which we have respecting the part played by quinine in originating *malarial hæmaturia*, we feel that in our treatment of such cases we have inaugurated an era of the most gratifying success.

We have been induced to pen the above crude thoughts because we have found this a new subject to many of my professional brethren, and because, as some one has said, "the aggregate of human experience has for its sum earthly wisdom."

PRESCOTT, ARK.

